# Syntheses and crystal structures of 2-(*p*-tol­yl)-1*H*-perimidine hemihydrate and 1-methyl-2-(*p*-tol­yl)-1*H*-perimidine

**DOI:** 10.1107/S2056989022000287

**Published:** 2022-01-14

**Authors:** Paulina Kalle, Sergei V. Tatarin, Marina A. Kiseleva, Alexander Yu. Zakharov, Daniil E. Smirnov, Stanislav I. Bezzubov

**Affiliations:** a N. S. Kurnakov Institute of General and Inorganic Chemistry, Russian Academy of Sciences, Leninsky pr. 31, Moscow 119991, Russian Federation; b Department of Chemistry, Lomonosov Moscow State University, Lenin’s Hills, 1-3, Moscow, 119991, Russian Federation

**Keywords:** crystal structure, perimidine, π–π stacking, hydrogen bonding, NMR study

## Abstract

The title compounds, 2-(4-methylphenyl)-1*H*-perimidine hemihydrate (**1**, C_18_H_14_N_2_·0.5H_2_O) and 1-methyl-2-(4-methylphenyl)-1*H*-perimidine (**2**, C_19_H_16_N_2_), were prepared and characterized by ^1^H NMR and single-crystal X-ray diffraction. The organic mol­ecule of the hemihydrate lies on a twofold rotation axis while the water mol­ecule lies on the inter­section of three twofold rotation axes (point group symmetry 222). As a consequence, the hydrogen atoms that are part of the N—H group and the water mol­ecule as well as the CH_3_ group of the *p*-tolyl ring are disordered over two positions. In compound **1**, the perimidine and the 2-aryl rings are slightly twisted while its *N*-methyl­ated derivative **2** has a more distorted conformation because of the steric repulsion between the *N*-methyl group and the 2-aryl ring. In the crystal structures, mol­ecules of perimidine **2** are held together only by C—H⋯π contacts while the parent perimidine **1** does not exhibit this type of inter­action. Its crystal packing is established by inter­molecular N—H⋯O hydrogen bonds with the solvent water mol­ecules and additionally stabilized by π–π stacking.

## Abstract 2

*N*-methyl­ation of the perimidine core results in an increase of the inter­planar angle between the perimidine and aryl rings. The crystal packing of the unsubstituted perimidine is formed by hydrogen bonding and π–π stacking while mol­ecules of its *N*-substituted analog are assembled by C—H⋯π inter­actions.

## Chemical context

Perimidines have found applications in industry as dyes and pigments because of their finely tunable optical properties (Pozharskii *et al.*, 2020 ▸). The introduction of electron-donating/withdrawing groups to the perimidine system dramatically affects its electronic structure and allows the color as well as color intensity of the perimidine to be varied. Additionally, a significant deepening of the color of perimidines can be achieved by decorating them with aromatic rings at position 2 while their optical characteristics can be modulated by varying the *N*-substituent (Sahiba & Agarwal, 2020 ▸). Recently, we have studied the effect of the *N*-substituent(s) on the structures of 2-(pyridin-2-yl)-1-*H*-perimidines (Kalle *et al.*, 2021 ▸). Herein, we report structural studies of 1-*H*-2-(*p*-tol­yl)-perimidine hemihydrate (**1**) and 1-methyl-2-(*p*-tol­yl)-perimidine (**2**). 
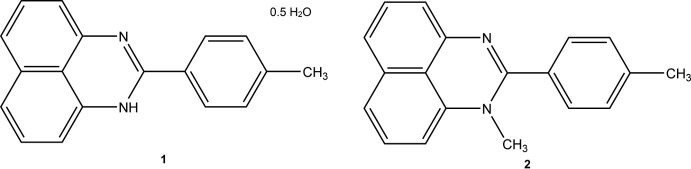


## Structural commentary

**Figure 1:**
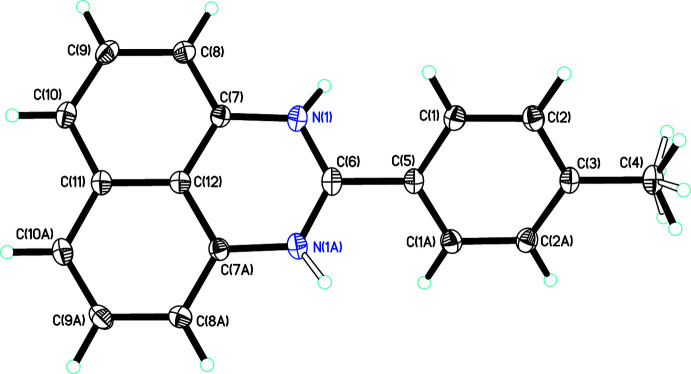
Mol­ecular structure of 1-*H*-2-(*p*-tol­yl)-perimidine (**1**), with displacement ellipsoids drawn at the 50% probability level. H atoms attached to N1 and N1*A* are present at half occupancy by virtue of the forced twofold symmetry. [Symmetry code: (A) −*x* + 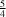 , −*y* + 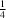 , *z*].

The perimidine mol­ecule of **1** possesses *C*2 symmetry with the twofold rotation axis passing through carbon atoms C3–C6, C11 and C12 (Fig. 1 ▸). This perimidine exhibits a C6—N1 bond length of 1.3345 (12) Å, a value inter­mediate between the average C—N single [1.366 (13) Å] and double [1.293 (11) Å] bond lengths in perimidines according to the Cambridge Crystal Structure Database (CSD version 5.43 November 2021; Groom *et al.*, 2016 ▸). The perimidine core of **1** is flat while the *p*-tolyl ring (C1–C5) forms a dihedral angle of 34.47 (5)° with the core, which is likely an effect of the crystal packing.

**Figure 2:**
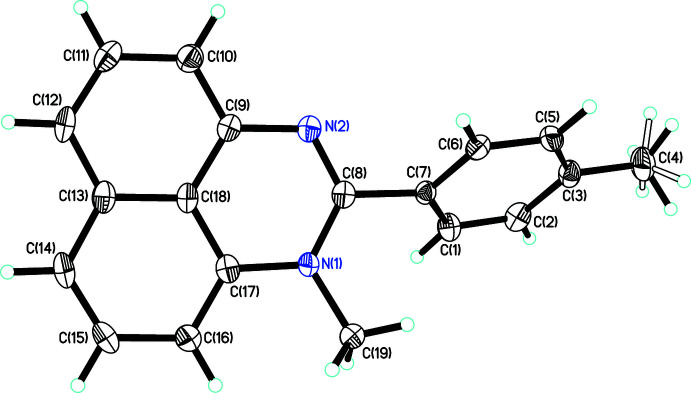
Mol­ecular structure of 1-methyl-2-(*p*-tol­yl)-perimidine (**2**), with displacement ellipsoids drawn at the 50% probability level.

The asymmetric unit of crystal **2** contains two mol­ecules, which are *N*-methyl­ated analogs of compound **1** (Fig. 2 ▸). Steric pressure exerted by the *N*-methyl group causes an increase of the inter­planar angle between the *p*-tolyl ring and the perimidine system [53.51 (10)° for one mol­ecule and 55.96 (9)° for the other]. Additionally, in the first mol­ecule, the angle between the N1—C19 bond and the centroid of the perimidine plane is as large as 8.7 (2)°. while the corresponding angle in the second mol­ecule is 6.1 (2)°.

## Supra­molecular features

**Figure 3:**
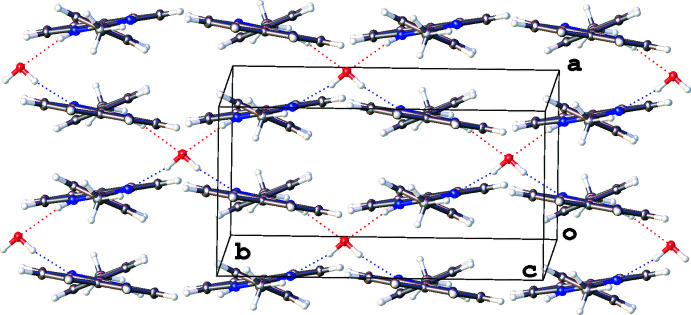
Hydrogen bonding and π—π stacking inter­actions in the crystal structure of 1-*H*-2-(*p*-tol­yl)-perimidine (**1**), with displacement ellipsoids drawn at the 50% probability level. Hydrogen atoms of the minor disorder component are omitted for clarity.

Recrystallization of **1** from toluene, di­chloro­methane, chloro­form or methanol gives crystals having an identical structure. An X-ray study of the crystals grown from hot toluene shows that compound **1** crystallizes as a hemihydrate in which the solvent mol­ecule plays a dominant role in the crystal packing. Each water mol­ecule, located at the inter­section of three twofold rotation axes (Wyckoff position 8*a*; point group symmetry 222), arranges four 2-(*p*-tol­yl)perimidines by mutual O—H⋯N and N—H⋯O inter­actions involving the O1 and N1 atoms as well as disordered hydrogen atoms H1 and H1*B* (Fig. 3 ▸, Table 1 ▸). These hydrogen-bonded associates containing the included water mol­ecule are additionally stabilized by π–π contacts between the aromatic units [*d*(C1⋯N1–C12_centroid_) = 3.3276 (11) Å, centroid–centroid shift of 1.591 (1) Å, *d*(C7⋯C1–C5_centroid_) = 3.5950 (11) Å, centroid–centroid shift of 1.433 (1) Å]. The same inter­actions combine the associates into infinite stacks along the *a* axis, forming two-dimensional structural arrays. The alignment of the arrays along the *c* axis by weak van der Waals inter­actions between perimidine C9—H9 and C10—H10 bonds and the methyl group (C4) of the *p*-tolyl ring completes the crystal packing of **1**.

**Figure 4:**
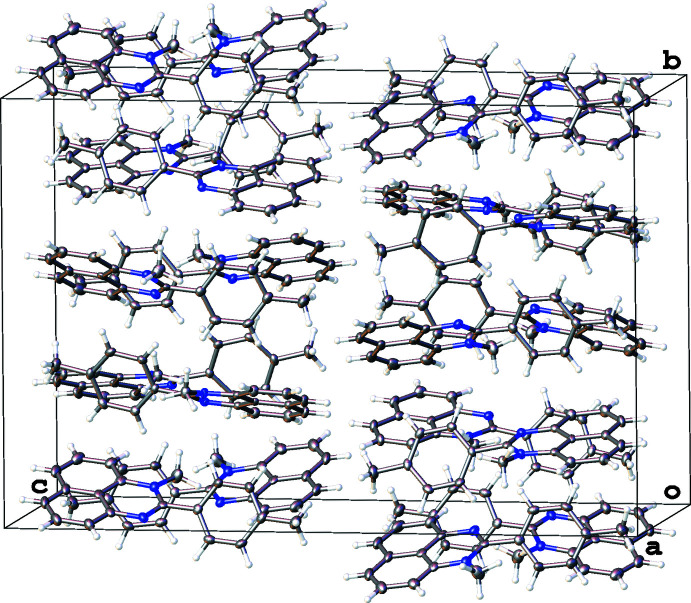
Fragment of the crystal packing of 1-methyl-2-(*p*-tol­yl)-perimidine (**2**), with displacement ellipsoids drawn at the 50% probability level. Hydrogen atoms of the minor parts of the disordered methyl groups are omitted for clarity.

In the crystal structure of **2** (Fig. 4 ▸), the two crystallographically independent mol­ecules are held together by C—H⋯π inter­actions between the *p*-tolyl and perimidine systems involving the H5 atom and the centroid of the C32–C37 ring [2.8226 (13) Å, 144.85 (18)°] and the H21 atom and the centroid of the C9–C13/C18 ring [2.6199 (12) Å, 145.74 (19)°]. The resulting dimers form stacks *via* similar non-covalent bonds involving the H24 atom and the centroid of the C9–C13/C18 ring [2.8676 (12) Å, 151.1 (2)°] and the H2 atom and the centroid of the C32–C37 ring [3.1727 (13) Å, 142.3 (2)°]. The resulting layers are grafted together by weak C—H⋯N contacts involving the H19*A* and N4 atoms [*d*(H⋯N) = 2.624 (2) Å, C—H⋯N angle = 166.86 (18)°], forming arrays in the *ab* plane. The three-dimensional crystal packing is organized by the alignment of the arrays along the *c* axis by weak van der Waals inter­actions in the same manner as in the crystal of **1**. It is inter­esting that compound **2**, in contrast to the parent perimidine **1**, crystallizes without notable π–π inter­actions.

## Database survey

A database search in the CSD (version 5.43 November 2021; Groom *et al.*, 2016 ▸) found only one crystal structure, a 2-aryl­perimidine hydrate in which one water mol­ecule combines two 2-(2-meth­oxy­phen­yl)-1-*H*-perimidines by O—H⋯N hydrogen bonds whereas the H atom at the second nitro­gen atom cannot inter­act with the oxygen atom of the water mol­ecule because it participates in an intra­molecular N—H⋯O bond with the meth­oxy group (PEKRIG; Foces-Foces *et al.*, 1993 ▸). A pseudo-tetra­hedral pattern of hydrogen-bonded organic mol­ecules around the included water mol­ecule is formed by 2-amino-4-(4-pyrid­yl)-6-phenyl­amino-1,3,5-triazine, which bears many more donor and acceptor hydrogen-bonding groups than compound **1** (TETRIT; Chan *et al.*, 1996 ▸). The crystal structures of organic hydrates including N—H⋯O inter­actions have also been published [KIJPUO (Black *et al.*, 1991 ▸); FAZRED (Rosling *et al.*, 1999 ▸)].

## Synthesis and crystallization

The title compounds were prepared as follows:

1-*H*-2-(*p*-tol­yl)perimidine (**1**).

A mixture of 1,8-di­aminona­phthalene (1.58 g, 0.01 mol), 4-methyl­benzaldehyde (1.18 ml, 0.01 mol) and sodium metabisulfite (5.7 g, 0.03 mol) in ethanol (40 ml) was refluxed under Ar for 2 h. The reaction mixture was cooled, filtered and the filtrate was evaporated to dryness and washed with water. The crude solid was recrystallized from toluene and dried *in vacuo*. Yield 2.20 g (85%). Single crystals suitable for X-ray analysis were grown from hot toluene.

^1^H NMR (DMSO-*d*_6_, ppm, 400 MHz): *δ* 2.34 (*s*, 3H, CH_3_), 6.65 (*d*, *J* = 7.2 Hz, 2H, H_naph_), 7.04 (*d*, *J* = 8.0 Hz, 2H, H_naph_), 7.15 (*t*, *J* = 7.2 Hz, 2H, H_naph_), 7.30 (*d*, *J* = 7.2 Hz, 2H, H_tol_), 7.95 (*d*, *J* = 8.0 Hz, 2H, H_tol_). See supplementary Fig. S1.

1-Methyl-2-(*p*-tol­yl)perimidine (**2**).

To a mixture of (**1**) (0.258 g, 1.0 mmol), solid KOH (0.056 g, 1.0 mmol) and anhydrous K_2_CO_3_ (0.138 g, 1.0 mmol) in anhydrous Ar-saturated aceto­nitrile methyl iodide (0.062 ml, 1.0 mmol) were added dropwise upon stirring and the resulting suspension was heated at 323 K for 1 h and then at room temperature for 1 h. The reaction mixture was evap­orated to dryness and the crude product was purified by column chromatography (eluent hexa­ne/ethyl acetate 1/1 → 1/5 *v*/*v*), recrystallized from a mixture of toluene/hexane and dried *in vacuo*. Yield 125 mg (46%). Single crystals suitable for X-ray analysis were grown by slow evaporation of the solvent from a solution of the substance in toluene.

^1^H NMR (CDCl_3_, ppm, 400 MHz): *δ* 2.42(*s*, 3H, CH_3_), 3.17 (*s*, 3H, N–CH_3_), 6.28 (*d*, *J* = 7.2 Hz, 1H, H_naph_), 6.94 (*d*, *J* = 7.3 Hz, 1H, H_naph_), 7.17–7.24 (*m*, 3H, H_naph_), 7.28–7.32 (*m*, 3H, H_naph_ + H_tol_), 7.44 (*d*, *J* = 7.7 Hz, 2H, H_tol_). See supplementary Fig. S2.

## Refinement

Crystal data, data collection and structure refinement details are summarized in Table 2 ▸. All C—H hydrogen atoms in the structures of **1** and **2** were placed in calculated positions and refined using a riding model [C—H = 0.94–0.97 Å with *U*_iso_(H) = 1.2–1.5*U*_eq_(C)]. N—H and O—H hydrogen atoms (structure **1**) were located in difference electron-density maps and were refined with a fixed occupancy of 0.5. *para*-Methyl groups in both crystallographically independent mol­ecules of **2** were found to be rotationally disordered with occupancy ratios of 0.6/0.4 and 0.7/0.3. The same group in the structure of **1** was similarly disordered with an occupancy ratio of 0.5/0.5. The SIMU instruction was used to restrain the *U*_ij_ components of the neighboring C6 and N1 atoms in the structure of **1**. The most disagreeable reflections with an error/s.u. of more than 10 (0 0 4 in the structure of **1**; 5 0 34 and 6 1 33 in the structure of **2**) were omitted using the OMIT instruction in *SHELXL* (Sheldrick, 2015 ▸).

## Supplementary Material

Crystal structure: contains datablock(s) 1, 2. DOI: 10.1107/S2056989022000287/wm5631sup1.cif

Structure factors: contains datablock(s) 1. DOI: 10.1107/S2056989022000287/wm56311sup6.hkl

Supporting information file. DOI: 10.1107/S2056989022000287/wm56311sup8.mol

Structure factors: contains datablock(s) 2. DOI: 10.1107/S2056989022000287/wm56312sup7.hkl

Supporting information file. DOI: 10.1107/S2056989022000287/wm56312sup9.mol

1H NMR for compound 1. DOI: 10.1107/S2056989022000287/wm5631sup4.pdf

1H NMR for compound 2. DOI: 10.1107/S2056989022000287/wm5631sup5.pdf

CCDC references: 2133148, 2133147

Additional supporting information:  crystallographic information; 3D view; checkCIF report

## Figures and Tables

**Table 1:** Hydrogen-bond geometry (Å, °) for **1** [Chem scheme1]

*D*—H⋯*A*	*D*—H	H⋯*A*	*D*⋯*A*	*D*—H⋯*A*
O1—H1*B*⋯N1	0.89 (3)	2.13 (3)	2.9826 (10)	162 (3)
N1—H1⋯O1	0.87 (3)	2.15 (3)	2.9826 (10)	160 (3)

**Table 2:** Experimental details

	C_18_H_14_N_2_·0.5H_2_O	C_19_H_16_N_2_
Crystal data		
*M*_r_	267.32	272.34
Crystal system, space group	Orthorhombic, *F* *d* *d* *d*	Orthorhombic, *P* *b* *c* *a*
Temperature (K)	100	100
*a*, *b*, *c* (Å)	7.2131 (2), 13.8648 (5), 53.4532 (18)	11.6878 (4), 18.0941 (6), 26.9604 (8)
*V* (Å^3^)	5345.8 (3)	5701.6 (3)
*Z*	16	16
Radiation type	Mo *K*α	Mo *K*α
μ (mm^−1^)	0.08	0.08
Crystal size (mm)	0.13 × 0.1 × 0.1	0.12 × 0.09 × 0.08

Data collection		
Diffractometer	Bruker D8 Venture	Bruker D8 Venture
Absorption correction	Multi-scan (*SADABS*; Krause *et al.*, 2015 ▸)	Multi-scan (*SADABS*; Krause *et al.*, 2015 ▸)
*T*_min_, *T*_max_	0.672, 0.746	0.676, 0.746
No. of measured, independent and observed [*I* > 2σ(*I*)] reflections	14708, 2138, 1630	53425, 5047, 3857
*R*_int_	0.044	0.093
(sin θ/λ)_max_ (Å^−1^)	0.725	0.596

Refinement		
*R*[*F* ^2^ > 2σ(*F* ^2^)], *wR*(*F* ^2^), *S*	0.049, 0.140, 1.04	0.073, 0.157, 1.14
No. of reflections	2138	5047
No. of parameters	126	383
No. of restraints	6	0
H-atom treatment	H atoms treated by a mixture of independent and constrained refinement	H-atom parameters constrained
Δρ_max_, Δρ_min_ (e Å^−3^)	0.42, −0.34	0.23, −0.29

Computer programs: *APEX3* and *SAINT* (Bruker, 2017 ▸), *SHELXS* (Sheldrick, 2008 ▸), *SHELXL* (Sheldrick, 2015 ▸) and *OLEX2* (Dolomanov *et al.*, 2009 ▸).

## supplementary crystallographic information

### 2-(4-Methylphenyl)-1H-perimidine hemihydrate (1) Crystal data

**Table:**

C_18_H_14_N_2_·0.5H_2_O	*D*_x_ = 1.329 Mg m^−^^3^
*M**_r_* = 267.32	Mo *K*α radiation, λ = 0.71073 Å
Orthorhombic, *F**d**d**d*	Cell parameters from 4300 reflections
*a* = 7.2131 (2) Å	θ = 3.0–31.5°
*b* = 13.8648 (5) Å	µ = 0.08 mm^−^^1^
*c* = 53.4532 (18) Å	*T* = 100 K
*V* = 5345.8 (3) Å^3^	Block, orange
*Z* = 16	0.13 × 0.1 × 0.1 mm
*F*(000) = 2256	

### 2-(4-Methylphenyl)-1H-perimidine hemihydrate (1) Data collection

**Table:**

Bruker D8 Venture diffractometer	2138 independent reflections
Radiation source: microfocus sealed X-ray tube, Incoatec Iµs	1630 reflections with *I* > 2σ(*I*)
Focusing mirrors monochromator	*R*_int_ = 0.044
Detector resolution: 10.4 pixels mm^-1^	θ_max_ = 31.0°, θ_min_ = 3.0°
ω–scan	*h* = −9→10
Absorption correction: multi-scan (*SADABS*; Krause *et al.*, 2015)	*k* = −20→16
*T*_min_ = 0.672, *T*_max_ = 0.746	*l* = −77→77
14708 measured reflections	

### 2-(4-Methylphenyl)-1H-perimidine hemihydrate (1) Refinement

**Table:**

Refinement on *F*^2^	Primary atom site location: structure-invariant direct methods
Least-squares matrix: full	Hydrogen site location: mixed
*R*[*F*^2^ > 2σ(*F*^2^)] = 0.049	H atoms treated by a mixture of independent and constrained refinement
*wR*(*F*^2^) = 0.140	*w* = 1/[σ^2^(*F*_o_^2^) + (0.0761*P*)^2^ + 4.7998*P*] where *P* = (*F*_o_^2^ + 2*F*_c_^2^)/3
*S* = 1.04	(Δ/σ)_max_ = 0.001
2138 reflections	Δρ_max_ = 0.42 e Å^−^^3^
126 parameters	Δρ_min_ = −0.34 e Å^−^^3^
6 restraints	

### 2-(4-Methylphenyl)-1H-perimidine hemihydrate (1) Special details

**Table:**

Geometry. All esds (except the esd in the dihedral angle between two l.s. planes) are estimated using the full covariance matrix. The cell esds are taken into account individually in the estimation of esds in distances, angles and torsion angles; correlations between esds in cell parameters are only used when they are defined by crystal symmetry. An approximate (isotropic) treatment of cell esds is used for estimating esds involving l.s. planes.

### 2-(4-Methylphenyl)-1H-perimidine hemihydrate (1) Fractional atomic coordinates and isotropic or equivalent isotropic displacement parameters (Å^2^)

**Table:**

	*x*	*y*	*z*	*U*_iso_*/*U*_eq_	Occ. (<1)
O1	0.8750	0.3750	0.3750	0.0216 (4)	
H1B	0.790 (4)	0.335 (2)	0.3693 (6)	0.036 (9)*	0.5
N1	0.65901 (13)	0.20824 (7)	0.35517 (2)	0.0176 (2)	
H1	0.708 (4)	0.253 (2)	0.3644 (6)	0.026 (8)*	0.5
C1	0.55996 (16)	0.20495 (8)	0.40781 (2)	0.0177 (2)	
H1A	0.508 (2)	0.2610 (11)	0.3988 (3)	0.024 (4)*	
C2	0.55826 (15)	0.20393 (8)	0.43382 (2)	0.0181 (3)	
H2	0.503 (2)	0.2585 (11)	0.4429 (3)	0.022 (3)*	
C3	0.6250	0.1250	0.44720 (3)	0.0171 (3)	
C4	0.6250	0.1250	0.47536 (3)	0.0226 (4)	
H4A	0.6649	0.1883	0.4815	0.034*	0.5
H4B	0.7105	0.0754	0.4815	0.034*	0.5
H4C	0.4996	0.1113	0.4815	0.034*	0.5
C5	0.6250	0.1250	0.39454 (3)	0.0155 (3)	
C6	0.6250	0.1250	0.36684 (3)	0.0182 (3)	
C7	0.65540 (15)	0.21186 (8)	0.32902 (2)	0.0148 (2)	
C8	0.68100 (16)	0.29750 (8)	0.31632 (2)	0.0188 (3)	
H8	0.705 (2)	0.3573 (10)	0.3255 (3)	0.022 (4)*	
C9	0.67686 (17)	0.29749 (8)	0.28998 (2)	0.0210 (3)	
H9	0.697 (2)	0.3594 (11)	0.2812 (3)	0.031 (4)*	
C10	0.65083 (16)	0.21420 (8)	0.27653 (2)	0.0193 (3)	
H10	0.654 (2)	0.2141 (10)	0.2581 (3)	0.029 (4)*	
C11	0.6250	0.1250	0.28908 (3)	0.0162 (3)	
C12	0.6250	0.1250	0.31562 (3)	0.0144 (3)	

### 2-(4-Methylphenyl)-1H-perimidine hemihydrate (1) Atomic displacement parameters (Å^2^)

**Table:**

	*U*^11^	*U*^22^	*U*^33^	*U*^12^	*U*^13^	*U*^23^
O1	0.0249 (9)	0.0176 (8)	0.0222 (8)	0.000	0.000	0.000
N1	0.0173 (4)	0.0223 (5)	0.0133 (4)	−0.0015 (3)	−0.0005 (3)	0.0017 (3)
C1	0.0172 (5)	0.0200 (5)	0.0160 (5)	−0.0012 (4)	0.0005 (4)	0.0013 (4)
C2	0.0172 (5)	0.0210 (5)	0.0160 (5)	−0.0009 (4)	0.0021 (4)	−0.0015 (4)
C3	0.0153 (7)	0.0230 (8)	0.0128 (7)	−0.0030 (6)	0.000	0.000
C4	0.0244 (8)	0.0294 (9)	0.0141 (7)	0.0001 (7)	0.000	0.000
C5	0.0126 (6)	0.0209 (7)	0.0130 (7)	−0.0034 (5)	0.000	0.000
C6	0.0150 (6)	0.0261 (7)	0.0134 (6)	−0.0007 (5)	0.000	0.000
C7	0.0140 (5)	0.0167 (5)	0.0137 (5)	−0.0007 (4)	−0.0005 (4)	0.0009 (4)
C8	0.0222 (5)	0.0163 (5)	0.0179 (5)	−0.0023 (4)	−0.0002 (4)	0.0010 (4)
C9	0.0251 (6)	0.0192 (5)	0.0188 (5)	−0.0008 (4)	0.0006 (4)	0.0060 (4)
C10	0.0202 (5)	0.0238 (6)	0.0140 (5)	0.0012 (4)	0.0003 (4)	0.0025 (4)
C11	0.0143 (7)	0.0201 (7)	0.0142 (7)	0.0011 (6)	0.000	0.000
C12	0.0116 (6)	0.0167 (7)	0.0149 (7)	0.0013 (5)	0.000	0.000

### 2-(4-Methylphenyl)-1H-perimidine hemihydrate (1) Geometric parameters (Å, º)

**Table:**

O1—H1B	0.89 (3)	C5—C1^i^	1.3971 (14)
N1—H1	0.87 (3)	C5—C6	1.481 (2)
N1—C6	1.3345 (12)	C6—N1^i^	1.3345 (12)
N1—C7	1.3993 (14)	C7—C8	1.3801 (15)
C1—H1A	0.987 (15)	C7—C12	1.4182 (13)
C1—C2	1.3901 (15)	C8—H8	0.978 (15)
C1—C5	1.3972 (14)	C8—C9	1.4083 (16)
C2—H2	0.983 (15)	C9—H9	0.988 (16)
C2—C3	1.3932 (14)	C9—C10	1.3734 (17)
C3—C2^i^	1.3932 (14)	C10—H10	0.987 (16)
C3—C4	1.505 (2)	C10—C11	1.4192 (13)
C4—H4A	0.9800	C11—C10^i^	1.4194 (13)
C4—H4B	0.9800	C11—C12	1.419 (2)
C4—H4C	0.9800	C12—C7^i^	1.4181 (13)
			
C6—N1—H1	116 (2)	N1^i^—C6—N1	124.28 (14)
C6—N1—C7	119.64 (10)	N1—C6—C5	117.86 (7)
C7—N1—H1	123 (2)	N1^i^—C6—C5	117.86 (7)
C2—C1—H1A	119.4 (9)	N1—C7—C12	118.49 (10)
C2—C1—C5	120.17 (11)	C8—C7—N1	121.32 (10)
C5—C1—H1A	120.3 (9)	C8—C7—C12	120.19 (10)
C1—C2—H2	119.4 (8)	C7—C8—H8	120.5 (9)
C1—C2—C3	121.22 (11)	C7—C8—C9	119.26 (11)
C3—C2—H2	119.3 (8)	C9—C8—H8	120.2 (9)
C2^i^—C3—C2	118.23 (14)	C8—C9—H9	118.1 (9)
C2^i^—C3—C4	120.88 (7)	C10—C9—C8	121.76 (11)
C2—C3—C4	120.89 (7)	C10—C9—H9	120.2 (9)
C3—C4—H4A	109.5	C9—C10—H10	121.5 (9)
C3—C4—H4B	109.5	C9—C10—C11	120.21 (11)
C3—C4—H4C	109.5	C11—C10—H10	118.3 (9)
H4A—C4—H4B	109.5	C10—C11—C10^i^	123.58 (14)
H4A—C4—H4C	109.5	C12—C11—C10	118.21 (7)
H4B—C4—H4C	109.5	C12—C11—C10^i^	118.21 (7)
C1^i^—C5—C1	118.98 (14)	C7^i^—C12—C7	119.34 (13)
C1^i^—C5—C6	120.51 (7)	C7—C12—C11	120.33 (7)
C1—C5—C6	120.51 (7)	C7^i^—C12—C11	120.33 (7)
			
N1—C7—C8—C9	179.77 (10)	C7—N1—C6—N1^i^	1.69 (7)
N1—C7—C12—C7^i^	1.61 (6)	C7—N1—C6—C5	−178.31 (7)
N1—C7—C12—C11	−178.39 (6)	C7—C8—C9—C10	−1.02 (18)
C1—C2—C3—C2^i^	−0.82 (7)	C8—C7—C12—C7^i^	−178.51 (12)
C1—C2—C3—C4	179.18 (7)	C8—C7—C12—C11	1.49 (12)
C1^i^—C5—C6—N1^i^	34.94 (7)	C8—C9—C10—C11	0.73 (17)
C1^i^—C5—C6—N1	−145.06 (7)	C9—C10—C11—C10^i^	−179.34 (13)
C1—C5—C6—N1	34.94 (7)	C9—C10—C11—C12	0.66 (12)
C1—C5—C6—N1^i^	−145.06 (7)	C10—C11—C12—C7	−1.75 (7)
C2—C1—C5—C1^i^	−0.81 (7)	C10^i^—C11—C12—C7^i^	−1.75 (7)
C2—C1—C5—C6	179.20 (7)	C10^i^—C11—C12—C7	178.25 (7)
C5—C1—C2—C3	1.64 (15)	C10—C11—C12—C7^i^	178.25 (7)
C6—N1—C7—C8	176.84 (9)	C12—C7—C8—C9	−0.10 (16)
C6—N1—C7—C12	−3.29 (13)		

Symmetry code: (i) −*x*+5/4, −*y*+1/4, *z*.

### 2-(4-Methylphenyl)-1H-perimidine hemihydrate (1) Hydrogen-bond geometry (Å, º)

**Table:**

*D*—H···*A*	*D*—H	H···*A*	*D*···*A*	*D*—H···*A*
O1—H1*B*···N1	0.89 (3)	2.13 (3)	2.9826 (10)	162 (3)
N1—H1···O1	0.87 (3)	2.15 (3)	2.9826 (10)	160 (3)

### 1-Methyl-2-(p-tolyl)-1-H-perimidine (2) Crystal data

**Table:**

C_19_H_16_N_2_	*D*_x_ = 1.269 Mg m^−^^3^
*M**_r_* = 272.34	Mo *K*α radiation, λ = 0.71073 Å
Orthorhombic, *P**b**c**a*	Cell parameters from 7540 reflections
*a* = 11.6878 (4) Å	θ = 2.2–28.3°
*b* = 18.0941 (6) Å	µ = 0.08 mm^−^^1^
*c* = 26.9604 (8) Å	*T* = 100 K
*V* = 5701.6 (3) Å^3^	Block, yellow
*Z* = 16	0.12 × 0.09 × 0.08 mm
*F*(000) = 2304	

### 1-Methyl-2-(p-tolyl)-1-H-perimidine (2) Data collection

**Table:**

Bruker D8 Venture diffractometer	5047 independent reflections
Radiation source: microfocus sealed X-ray tube, Incoatec Iµs	3857 reflections with *I* > 2σ(*I*)
Focusing mirrors monochromator	*R*_int_ = 0.093
Detector resolution: 10.4 pixels mm^-1^	θ_max_ = 25.1°, θ_min_ = 2.2°
ω–scan	*h* = −13→13
Absorption correction: multi-scan (*SADABS*; Krause *et al.*, 2015)	*k* = −21→21
*T*_min_ = 0.676, *T*_max_ = 0.746	*l* = −31→32
53425 measured reflections	

### 1-Methyl-2-(p-tolyl)-1-H-perimidine (2) Refinement

**Table:**

Refinement on *F*^2^	Primary atom site location: dual
Least-squares matrix: full	Hydrogen site location: inferred from neighbouring sites
*R*[*F*^2^ > 2σ(*F*^2^)] = 0.073	H-atom parameters constrained
*wR*(*F*^2^) = 0.157	*w* = 1/[σ^2^(*F*_o_^2^) + (0.0485*P*)^2^ + 7.2287*P*] where *P* = (*F*_o_^2^ + 2*F*_c_^2^)/3
*S* = 1.14	(Δ/σ)_max_ < 0.001
5047 reflections	Δρ_max_ = 0.23 e Å^−^^3^
383 parameters	Δρ_min_ = −0.29 e Å^−^^3^
0 restraints	

### 1-Methyl-2-(p-tolyl)-1-H-perimidine (2) Special details

**Table:**

Geometry. All esds (except the esd in the dihedral angle between two l.s. planes) are estimated using the full covariance matrix. The cell esds are taken into account individually in the estimation of esds in distances, angles and torsion angles; correlations between esds in cell parameters are only used when they are defined by crystal symmetry. An approximate (isotropic) treatment of cell esds is used for estimating esds involving l.s. planes.

### 1-Methyl-2-(p-tolyl)-1-H-perimidine (2) Fractional atomic coordinates and isotropic or equivalent isotropic displacement parameters (Å^2^)

**Table:**

	*x*	*y*	*z*	*U*_iso_*/*U*_eq_	Occ. (<1)
N1	0.43727 (19)	0.18405 (12)	0.31230 (8)	0.0175 (5)	
N2	0.2447 (2)	0.21911 (12)	0.29690 (8)	0.0185 (5)	
C1	0.3882 (2)	0.11253 (15)	0.21059 (10)	0.0209 (6)	
H1	0.4128	0.0760	0.2335	0.025*	
C2	0.3842 (3)	0.09595 (15)	0.16034 (11)	0.0233 (7)	
H2	0.4038	0.0477	0.1493	0.028*	
C3	0.3516 (2)	0.14950 (16)	0.12585 (10)	0.0217 (7)	
C4	0.3475 (3)	0.13008 (18)	0.07119 (11)	0.0325 (8)	
H4AA	0.4255	0.1230	0.0588	0.049*	0.7
H4AB	0.3107	0.1703	0.0528	0.049*	0.7
H4AC	0.3036	0.0844	0.0667	0.049*	0.7
H4BD	0.2695	0.1372	0.0587	0.049*	0.3
H4BE	0.3702	0.0784	0.0667	0.049*	0.3
H4BF	0.4002	0.1622	0.0528	0.049*	0.3
C5	0.3240 (2)	0.21969 (16)	0.14290 (10)	0.0224 (7)	
H5	0.3035	0.2571	0.1199	0.027*	
C6	0.3261 (2)	0.23577 (15)	0.19325 (10)	0.0197 (6)	
H6	0.3066	0.2840	0.2043	0.024*	
C7	0.3567 (2)	0.18187 (15)	0.22769 (10)	0.0166 (6)	
C8	0.3451 (2)	0.19748 (14)	0.28191 (10)	0.0167 (6)	
C9	0.2275 (2)	0.22857 (14)	0.34790 (10)	0.0175 (6)	
C10	0.1229 (3)	0.25252 (16)	0.36522 (11)	0.0239 (7)	
H10	0.0632	0.2635	0.3425	0.029*	
C11	0.1044 (3)	0.26077 (16)	0.41622 (11)	0.0270 (7)	
H11	0.0322	0.2779	0.4277	0.032*	
C12	0.1890 (3)	0.24445 (16)	0.45006 (11)	0.0258 (7)	
H12	0.1742	0.2496	0.4845	0.031*	
C13	0.2973 (3)	0.22019 (15)	0.43391 (10)	0.0215 (7)	
C14	0.3895 (3)	0.20256 (16)	0.46636 (11)	0.0268 (7)	
H14	0.3798	0.2075	0.5012	0.032*	
C15	0.4915 (3)	0.17867 (16)	0.44797 (11)	0.0261 (7)	
H15	0.5510	0.1663	0.4705	0.031*	
C16	0.5117 (3)	0.17171 (16)	0.39662 (10)	0.0233 (7)	
H16	0.5840	0.1556	0.3846	0.028*	
C17	0.4243 (2)	0.18870 (14)	0.36411 (10)	0.0184 (6)	
C18	0.3162 (2)	0.21197 (14)	0.38191 (10)	0.0174 (6)	
C19	0.5531 (2)	0.17366 (16)	0.29268 (10)	0.0229 (7)	
H19A	0.5765	0.1221	0.2973	0.034*	
H19B	0.6063	0.2062	0.3104	0.034*	
H19C	0.5540	0.1857	0.2572	0.034*	
N3	0.2012 (2)	0.42161 (12)	0.21218 (8)	0.0219 (6)	
N4	0.3830 (2)	0.47987 (13)	0.20977 (8)	0.0226 (6)	
C20	0.2838 (3)	0.40519 (16)	0.32035 (10)	0.0259 (7)	
H20	0.2647	0.3582	0.3071	0.031*	
C21	0.2985 (3)	0.41308 (16)	0.37083 (11)	0.0287 (7)	
H21	0.2901	0.3711	0.3917	0.034*	
C22	0.3253 (3)	0.48092 (16)	0.39184 (11)	0.0258 (7)	
C23	0.3434 (3)	0.48919 (19)	0.44674 (11)	0.0387 (9)	
H23A	0.4250	0.4843	0.4543	0.058*	0.6
H23B	0.3005	0.4507	0.4643	0.058*	0.6
H23C	0.3164	0.5379	0.4574	0.058*	0.6
H23D	0.3890	0.5336	0.4532	0.058*	0.4
H23E	0.3838	0.4457	0.4595	0.058*	0.4
H23F	0.2691	0.4936	0.4633	0.058*	0.4
C24	0.3369 (3)	0.54109 (16)	0.36007 (11)	0.0255 (7)	
H24	0.3546	0.5883	0.3735	0.031*	
C25	0.3231 (3)	0.53364 (15)	0.30924 (11)	0.0238 (7)	
H25	0.3318	0.5756	0.2884	0.029*	
C26	0.2967 (2)	0.46554 (15)	0.28853 (10)	0.0205 (6)	
C27	0.2939 (3)	0.45635 (14)	0.23366 (10)	0.0208 (7)	
C28	0.3886 (3)	0.46785 (15)	0.15836 (11)	0.0250 (7)	
C29	0.4843 (3)	0.49011 (17)	0.13268 (11)	0.0291 (7)	
H29	0.5461	0.5130	0.1496	0.035*	
C30	0.4898 (3)	0.47866 (17)	0.08107 (12)	0.0359 (8)	
H30	0.5562	0.4935	0.0634	0.043*	
C31	0.4011 (3)	0.44640 (17)	0.05601 (11)	0.0347 (8)	
H31	0.4064	0.4400	0.0211	0.042*	
C32	0.3017 (3)	0.42245 (16)	0.08105 (11)	0.0300 (8)	
C33	0.2067 (3)	0.38821 (17)	0.05775 (12)	0.0350 (8)	
H33	0.2070	0.3814	0.0228	0.042*	
C34	0.1150 (3)	0.36494 (17)	0.08484 (12)	0.0356 (8)	
H34	0.0530	0.3414	0.0684	0.043*	
C35	0.1098 (3)	0.37489 (16)	0.13666 (11)	0.0291 (7)	
H35	0.0449	0.3586	0.1549	0.035*	
C36	0.2001 (3)	0.40848 (15)	0.16054 (11)	0.0241 (7)	
C37	0.2970 (3)	0.43318 (15)	0.13339 (11)	0.0241 (7)	
C38	0.0958 (3)	0.40401 (19)	0.23934 (12)	0.0341 (8)	
H38A	0.0888	0.3503	0.2429	0.051*	
H38B	0.0297	0.4231	0.2210	0.051*	
H38C	0.0985	0.4269	0.2723	0.051*	

### 1-Methyl-2-(p-tolyl)-1-H-perimidine (2) Atomic displacement parameters (Å^2^)

**Table:**

	*U*^11^	*U*^22^	*U*^33^	*U*^12^	*U*^13^	*U*^23^
N1	0.0187 (13)	0.0200 (12)	0.0137 (12)	0.0014 (10)	0.0004 (10)	−0.0016 (9)
N2	0.0223 (13)	0.0151 (12)	0.0180 (13)	−0.0003 (10)	0.0015 (10)	−0.0008 (9)
C1	0.0273 (17)	0.0156 (14)	0.0198 (16)	−0.0042 (12)	0.0009 (13)	0.0021 (11)
C2	0.0266 (17)	0.0176 (14)	0.0257 (17)	−0.0053 (13)	0.0035 (13)	−0.0053 (12)
C3	0.0216 (16)	0.0276 (16)	0.0159 (15)	−0.0070 (13)	−0.0003 (12)	−0.0027 (12)
C4	0.041 (2)	0.0383 (19)	0.0184 (17)	−0.0029 (15)	−0.0007 (14)	−0.0038 (13)
C5	0.0211 (16)	0.0264 (16)	0.0195 (16)	−0.0002 (13)	−0.0001 (12)	0.0064 (12)
C6	0.0192 (15)	0.0192 (15)	0.0207 (16)	0.0012 (12)	0.0038 (12)	−0.0001 (11)
C7	0.0160 (15)	0.0199 (14)	0.0140 (14)	−0.0024 (11)	0.0005 (11)	−0.0023 (11)
C8	0.0225 (16)	0.0095 (13)	0.0180 (15)	−0.0037 (11)	0.0016 (12)	0.0003 (10)
C9	0.0215 (16)	0.0121 (13)	0.0188 (15)	−0.0024 (11)	0.0030 (12)	−0.0007 (10)
C10	0.0232 (17)	0.0254 (16)	0.0232 (16)	−0.0003 (13)	0.0019 (13)	−0.0006 (12)
C11	0.0258 (17)	0.0288 (17)	0.0265 (17)	0.0009 (14)	0.0100 (14)	−0.0015 (13)
C12	0.0351 (19)	0.0268 (16)	0.0155 (15)	−0.0041 (14)	0.0084 (13)	−0.0047 (12)
C13	0.0302 (17)	0.0170 (14)	0.0174 (15)	−0.0040 (13)	0.0038 (13)	0.0000 (11)
C14	0.038 (2)	0.0294 (17)	0.0128 (15)	−0.0052 (15)	−0.0012 (13)	−0.0009 (12)
C15	0.0322 (18)	0.0278 (16)	0.0182 (16)	−0.0028 (14)	−0.0082 (14)	0.0014 (12)
C16	0.0228 (16)	0.0260 (16)	0.0210 (16)	0.0011 (13)	−0.0015 (13)	−0.0012 (12)
C17	0.0254 (16)	0.0123 (14)	0.0174 (15)	−0.0021 (12)	−0.0005 (12)	−0.0009 (11)
C18	0.0246 (16)	0.0131 (13)	0.0146 (14)	−0.0061 (12)	0.0023 (12)	−0.0013 (11)
C19	0.0190 (15)	0.0301 (17)	0.0196 (15)	0.0008 (12)	0.0004 (12)	−0.0015 (12)
N3	0.0266 (14)	0.0194 (12)	0.0197 (13)	0.0025 (11)	0.0023 (11)	−0.0007 (10)
N4	0.0283 (15)	0.0201 (12)	0.0195 (14)	0.0027 (11)	0.0024 (11)	0.0046 (10)
C20	0.039 (2)	0.0180 (15)	0.0203 (16)	−0.0046 (13)	0.0070 (14)	−0.0023 (12)
C21	0.042 (2)	0.0235 (16)	0.0202 (17)	−0.0018 (14)	0.0080 (14)	0.0013 (12)
C22	0.0284 (18)	0.0270 (16)	0.0219 (16)	0.0017 (13)	0.0026 (13)	−0.0063 (12)
C23	0.057 (2)	0.0337 (19)	0.0251 (18)	0.0013 (17)	−0.0003 (17)	−0.0079 (14)
C24	0.0319 (18)	0.0169 (15)	0.0276 (18)	−0.0003 (13)	0.0002 (14)	−0.0065 (12)
C25	0.0249 (17)	0.0168 (15)	0.0297 (18)	−0.0004 (12)	0.0013 (13)	0.0026 (12)
C26	0.0191 (15)	0.0223 (15)	0.0201 (16)	0.0023 (12)	0.0034 (12)	−0.0021 (12)
C27	0.0294 (17)	0.0112 (14)	0.0219 (16)	0.0050 (12)	0.0008 (13)	0.0016 (11)
C28	0.0374 (19)	0.0171 (15)	0.0205 (16)	0.0083 (13)	0.0045 (14)	0.0051 (12)
C29	0.036 (2)	0.0277 (17)	0.0234 (17)	0.0025 (14)	0.0060 (14)	0.0052 (13)
C30	0.051 (2)	0.0285 (18)	0.0279 (19)	0.0061 (16)	0.0166 (17)	0.0078 (14)
C31	0.062 (3)	0.0271 (17)	0.0153 (16)	0.0097 (17)	0.0064 (16)	0.0034 (13)
C32	0.052 (2)	0.0214 (16)	0.0167 (16)	0.0119 (15)	0.0012 (15)	0.0031 (12)
C33	0.060 (2)	0.0280 (18)	0.0176 (17)	0.0130 (17)	−0.0071 (16)	0.0014 (13)
C34	0.049 (2)	0.0280 (18)	0.0299 (19)	0.0111 (16)	−0.0146 (17)	−0.0047 (14)
C35	0.0351 (19)	0.0222 (16)	0.0299 (18)	0.0060 (14)	−0.0040 (15)	−0.0024 (13)
C36	0.0338 (18)	0.0167 (14)	0.0218 (16)	0.0103 (13)	−0.0037 (14)	0.0014 (12)
C37	0.0372 (19)	0.0134 (14)	0.0217 (16)	0.0105 (13)	−0.0004 (14)	0.0043 (11)
C38	0.0299 (19)	0.041 (2)	0.0308 (19)	−0.0058 (15)	0.0084 (15)	−0.0078 (14)

### 1-Methyl-2-(p-tolyl)-1-H-perimidine (2) Geometric parameters (Å, º)

**Table:**

N1—C8	1.375 (3)	N3—C27	1.380 (4)
N1—C17	1.407 (3)	N3—C36	1.412 (4)
N1—C19	1.466 (4)	N3—C38	1.468 (4)
N2—C8	1.301 (4)	N4—C27	1.297 (4)
N2—C9	1.400 (3)	N4—C28	1.404 (4)
C1—H1	0.9500	C20—H20	0.9500
C1—C2	1.388 (4)	C20—C21	1.379 (4)
C1—C7	1.386 (4)	C20—C26	1.397 (4)
C2—H2	0.9500	C21—H21	0.9500
C2—C3	1.396 (4)	C21—C22	1.388 (4)
C3—C4	1.516 (4)	C22—C23	1.503 (4)
C3—C5	1.389 (4)	C22—C24	1.392 (4)
C4—H4AA	0.9800	C23—H23A	0.9800
C4—H4AB	0.9800	C23—H23B	0.9800
C4—H4AC	0.9800	C23—H23C	0.9800
C4—H4BD	0.9800	C23—H23D	0.9800
C4—H4BE	0.9800	C23—H23E	0.9800
C4—H4BF	0.9800	C23—H23F	0.9800
C5—H5	0.9500	C24—H24	0.9500
C5—C6	1.388 (4)	C24—C25	1.386 (4)
C6—H6	0.9500	C25—H25	0.9500
C6—C7	1.393 (4)	C25—C26	1.388 (4)
C7—C8	1.495 (4)	C26—C27	1.489 (4)
C9—C10	1.378 (4)	C28—C29	1.376 (4)
C9—C18	1.417 (4)	C28—C37	1.412 (4)
C10—H10	0.9500	C29—H29	0.9500
C10—C11	1.400 (4)	C29—C30	1.408 (4)
C11—H11	0.9500	C30—H30	0.9500
C11—C12	1.378 (4)	C30—C31	1.369 (5)
C12—H12	0.9500	C31—H31	0.9500
C12—C13	1.409 (4)	C31—C32	1.412 (5)
C13—C14	1.424 (4)	C32—C33	1.418 (5)
C13—C18	1.427 (4)	C32—C37	1.425 (4)
C14—H14	0.9500	C33—H33	0.9500
C14—C15	1.361 (4)	C33—C34	1.364 (5)
C15—H15	0.9500	C34—H34	0.9500
C15—C16	1.410 (4)	C34—C35	1.410 (4)
C16—H16	0.9500	C35—H35	0.9500
C16—C17	1.381 (4)	C35—C36	1.377 (4)
C17—C18	1.416 (4)	C36—C37	1.421 (4)
C19—H19A	0.9800	C38—H38A	0.9800
C19—H19B	0.9800	C38—H38B	0.9800
C19—H19C	0.9800	C38—H38C	0.9800
			
C8—N1—C17	119.8 (2)	C27—N3—C36	119.8 (3)
C8—N1—C19	122.1 (2)	C27—N3—C38	123.2 (2)
C17—N1—C19	117.7 (2)	C36—N3—C38	116.6 (3)
C8—N2—C9	118.1 (2)	C27—N4—C28	118.5 (3)
C2—C1—H1	119.6	C21—C20—H20	119.6
C7—C1—H1	119.6	C21—C20—C26	120.8 (3)
C7—C1—C2	120.7 (3)	C26—C20—H20	119.6
C1—C2—H2	119.7	C20—C21—H21	119.2
C1—C2—C3	120.6 (3)	C20—C21—C22	121.5 (3)
C3—C2—H2	119.7	C22—C21—H21	119.2
C2—C3—C4	119.7 (3)	C21—C22—C23	121.5 (3)
C5—C3—C2	118.5 (3)	C21—C22—C24	117.6 (3)
C5—C3—C4	121.8 (3)	C24—C22—C23	121.0 (3)
C3—C4—H4AA	109.5	C22—C23—H23A	109.5
C3—C4—H4AB	109.5	C22—C23—H23B	109.5
C3—C4—H4AC	109.5	C22—C23—H23C	109.5
C3—C4—H4BD	109.5	C22—C23—H23D	109.5
C3—C4—H4BE	109.5	C22—C23—H23E	109.5
C3—C4—H4BF	109.5	C22—C23—H23F	109.5
H4AA—C4—H4AB	109.5	H23A—C23—H23B	109.5
H4AA—C4—H4AC	109.5	H23A—C23—H23C	109.5
H4AB—C4—H4AC	109.5	H23B—C23—H23C	109.5
H4BD—C4—H4BE	109.5	H23D—C23—H23E	109.5
H4BD—C4—H4BF	109.5	H23D—C23—H23F	109.5
H4BE—C4—H4BF	109.5	H23E—C23—H23F	109.5
C3—C5—H5	119.6	C22—C24—H24	119.3
C6—C5—C3	120.7 (3)	C25—C24—C22	121.4 (3)
C6—C5—H5	119.6	C25—C24—H24	119.3
C5—C6—H6	119.7	C24—C25—H25	119.7
C5—C6—C7	120.6 (3)	C24—C25—C26	120.7 (3)
C7—C6—H6	119.7	C26—C25—H25	119.7
C1—C7—C6	118.7 (2)	C20—C26—C27	121.4 (2)
C1—C7—C8	121.3 (2)	C25—C26—C20	118.1 (3)
C6—C7—C8	119.7 (2)	C25—C26—C27	120.3 (3)
N1—C8—C7	118.6 (2)	N3—C27—C26	119.0 (3)
N2—C8—N1	125.1 (2)	N4—C27—N3	124.9 (3)
N2—C8—C7	116.2 (2)	N4—C27—C26	116.0 (3)
N2—C9—C18	120.3 (2)	N4—C28—C37	120.3 (3)
C10—C9—N2	119.9 (3)	C29—C28—N4	119.3 (3)
C10—C9—C18	119.8 (2)	C29—C28—C37	120.4 (3)
C9—C10—H10	119.9	C28—C29—H29	120.3
C9—C10—C11	120.3 (3)	C28—C29—C30	119.5 (3)
C11—C10—H10	119.9	C30—C29—H29	120.3
C10—C11—H11	119.5	C29—C30—H30	119.5
C12—C11—C10	121.1 (3)	C31—C30—C29	121.0 (3)
C12—C11—H11	119.5	C31—C30—H30	119.5
C11—C12—H12	119.8	C30—C31—H31	119.4
C11—C12—C13	120.5 (3)	C30—C31—C32	121.2 (3)
C13—C12—H12	119.8	C32—C31—H31	119.4
C12—C13—C14	124.0 (3)	C31—C32—C33	124.5 (3)
C12—C13—C18	118.4 (3)	C31—C32—C37	117.6 (3)
C14—C13—C18	117.6 (3)	C33—C32—C37	117.9 (3)
C13—C14—H14	119.7	C32—C33—H33	119.6
C15—C14—C13	120.7 (3)	C34—C33—C32	120.9 (3)
C15—C14—H14	119.7	C34—C33—H33	119.6
C14—C15—H15	118.9	C33—C34—H34	119.2
C14—C15—C16	122.2 (3)	C33—C34—C35	121.7 (3)
C16—C15—H15	118.9	C35—C34—H34	119.2
C15—C16—H16	120.7	C34—C35—H35	120.4
C17—C16—C15	118.7 (3)	C36—C35—C34	119.1 (3)
C17—C16—H16	120.7	C36—C35—H35	120.4
N1—C17—C18	116.8 (2)	N3—C36—C37	116.6 (3)
C16—C17—N1	122.5 (3)	C35—C36—N3	122.8 (3)
C16—C17—C18	120.7 (3)	C35—C36—C37	120.6 (3)
C9—C18—C13	120.0 (3)	C28—C37—C32	120.2 (3)
C17—C18—C9	119.8 (2)	C28—C37—C36	119.9 (3)
C17—C18—C13	120.1 (3)	C36—C37—C32	119.9 (3)
N1—C19—H19A	109.5	N3—C38—H38A	109.5
N1—C19—H19B	109.5	N3—C38—H38B	109.5
N1—C19—H19C	109.5	N3—C38—H38C	109.5
H19A—C19—H19B	109.5	H38A—C38—H38B	109.5
H19A—C19—H19C	109.5	H38A—C38—H38C	109.5
H19B—C19—H19C	109.5	H38B—C38—H38C	109.5
			
N1—C17—C18—C9	0.5 (4)	N3—C36—C37—C28	0.9 (4)
N1—C17—C18—C13	−178.1 (2)	N3—C36—C37—C32	−179.5 (2)
N2—C9—C10—C11	178.8 (3)	N4—C28—C29—C30	−179.5 (3)
N2—C9—C18—C13	−178.6 (2)	N4—C28—C37—C32	178.7 (2)
N2—C9—C18—C17	2.7 (4)	N4—C28—C37—C36	−1.7 (4)
C1—C2—C3—C4	−179.8 (3)	C20—C21—C22—C23	−178.9 (3)
C1—C2—C3—C5	0.4 (4)	C20—C21—C22—C24	0.0 (5)
C1—C7—C8—N1	56.1 (4)	C20—C26—C27—N3	54.3 (4)
C1—C7—C8—N2	−120.2 (3)	C20—C26—C27—N4	−123.2 (3)
C2—C1—C7—C6	−3.0 (4)	C21—C20—C26—C25	−0.9 (5)
C2—C1—C7—C8	171.5 (3)	C21—C20—C26—C27	173.2 (3)
C2—C3—C5—C6	−1.5 (4)	C21—C22—C24—C25	−0.5 (5)
C3—C5—C6—C7	0.4 (4)	C22—C24—C25—C26	0.2 (5)
C4—C3—C5—C6	178.7 (3)	C23—C22—C24—C25	178.4 (3)
C5—C6—C7—C1	1.9 (4)	C24—C25—C26—C20	0.4 (4)
C5—C6—C7—C8	−172.8 (3)	C24—C25—C26—C27	−173.7 (3)
C6—C7—C8—N1	−129.4 (3)	C25—C26—C27—N3	−131.8 (3)
C6—C7—C8—N2	54.3 (4)	C25—C26—C27—N4	50.7 (4)
C7—C1—C2—C3	2.0 (4)	C26—C20—C21—C22	0.7 (5)
C8—N1—C17—C16	176.2 (2)	C27—N3—C36—C35	−179.2 (3)
C8—N1—C17—C18	−3.9 (4)	C27—N3—C36—C37	−0.5 (4)
C8—N2—C9—C10	179.0 (3)	C27—N4—C28—C29	−177.7 (3)
C8—N2—C9—C18	−2.5 (4)	C27—N4—C28—C37	2.1 (4)
C9—N2—C8—N1	−1.1 (4)	C28—N4—C27—N3	−1.8 (4)
C9—N2—C8—C7	174.9 (2)	C28—N4—C27—C26	175.5 (2)
C9—C10—C11—C12	−0.8 (4)	C28—C29—C30—C31	0.7 (5)
C10—C9—C18—C13	−0.1 (4)	C29—C28—C37—C32	−1.5 (4)
C10—C9—C18—C17	−178.8 (3)	C29—C28—C37—C36	178.1 (3)
C10—C11—C12—C13	1.1 (4)	C29—C30—C31—C32	−1.0 (5)
C11—C12—C13—C14	179.7 (3)	C30—C31—C32—C33	−179.6 (3)
C11—C12—C13—C18	−0.9 (4)	C30—C31—C32—C37	0.1 (4)
C12—C13—C14—C15	179.3 (3)	C31—C32—C33—C34	178.3 (3)
C12—C13—C18—C9	0.5 (4)	C31—C32—C37—C28	1.1 (4)
C12—C13—C18—C17	179.1 (3)	C31—C32—C37—C36	−178.5 (3)
C13—C14—C15—C16	1.3 (5)	C32—C33—C34—C35	1.0 (5)
C14—C13—C18—C9	179.9 (2)	C33—C32—C37—C28	−179.2 (3)
C14—C13—C18—C17	−1.4 (4)	C33—C32—C37—C36	1.2 (4)
C14—C15—C16—C17	−1.0 (4)	C33—C34—C35—C36	−0.5 (5)
C15—C16—C17—N1	179.3 (2)	C34—C35—C36—N3	179.0 (3)
C15—C16—C17—C18	−0.6 (4)	C34—C35—C36—C37	0.3 (4)
C16—C17—C18—C9	−179.5 (2)	C35—C36—C37—C28	179.7 (3)
C16—C17—C18—C13	1.8 (4)	C35—C36—C37—C32	−0.7 (4)
C17—N1—C8—N2	4.4 (4)	C36—N3—C27—N4	1.0 (4)
C17—N1—C8—C7	−171.5 (2)	C36—N3—C27—C26	−176.3 (2)
C18—C9—C10—C11	0.3 (4)	C37—C28—C29—C30	0.6 (4)
C18—C13—C14—C15	−0.1 (4)	C37—C32—C33—C34	−1.4 (4)
C19—N1—C8—N2	−168.0 (3)	C38—N3—C27—N4	−172.2 (3)
C19—N1—C8—C7	16.1 (4)	C38—N3—C27—C26	10.6 (4)
C19—N1—C17—C16	−11.1 (4)	C38—N3—C36—C35	−5.7 (4)
C19—N1—C17—C18	168.9 (2)	C38—N3—C36—C37	173.1 (3)
