# Supplementary figures and images for: Syntheses and crystal structures of 2-(p-tol­yl)-1H-perimidine hemihydrate and 1-methyl-2-(p-tol­yl)-1H-perimidine
Source: Acta Crystallogr E Crystallogr Commun. 2022 Jan 14;78(Pt 2):169–72. doi: 10.1107/S2056989022000287 (PMC8819453; doi:10.1107/S2056989022000287)

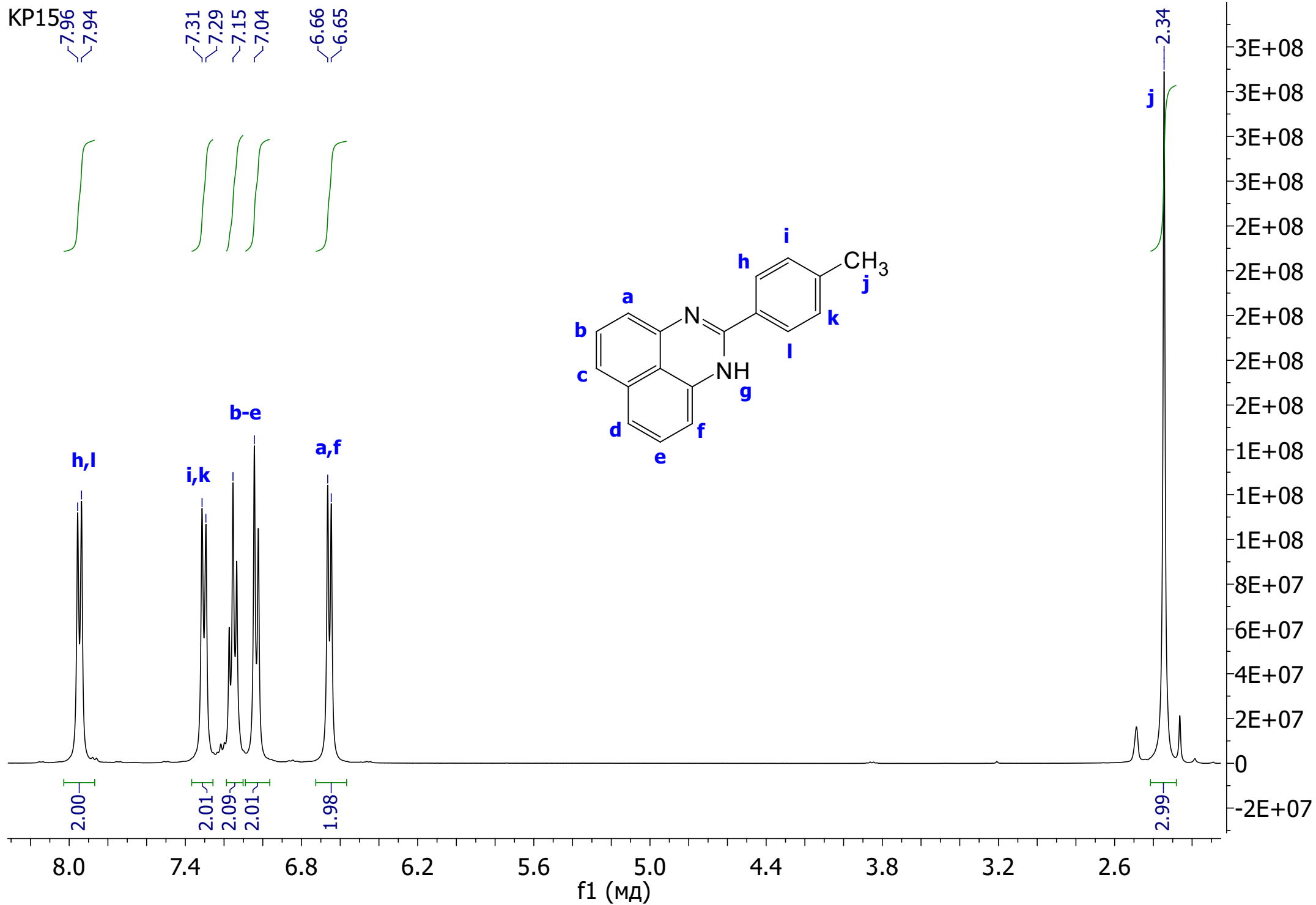

Supplement: Supplementary file 6 [file e-78-00169-sup4.pdf]

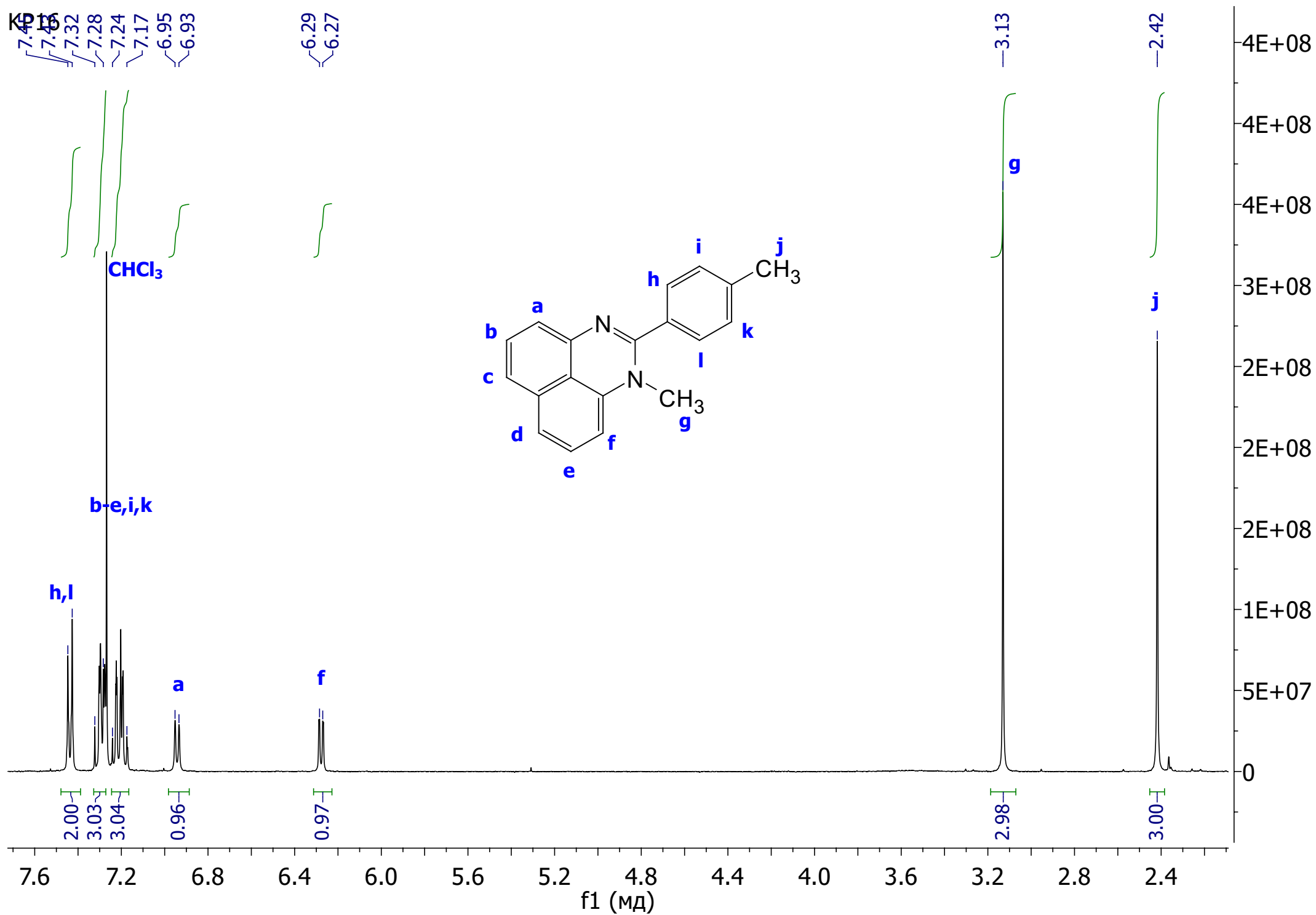

Supplement: Supplementary file 7 [file e-78-00169-sup5.pdf]
